# Chicago Medical Society

**Published:** 1887-11

**Authors:** 


### Society Reports.

### CHICAGO MEDICAL SOCIETY.

Stated Meeting, June 6, 1887.

The President, Wm. T. Belfield, M. D., in the chair.

Dr. Frank Billings exhibited a very interesting collection of

### BACTERIAL CULTURES, and explained the mode of their culture, and gave the name and description of each bacterium.

The President asked whether this method of culture was not of extreme value in differentiating the different sorts of bacteria one from another, and whether this did not enable investigators to distinguish the different sorts of bacteria.

Dr. F. Billings: At the present time there are three comma bacilli that are well known: the comma bacillus of Asiatic cholera, discovered by Koch, the one discovered by Deneke in old cheese, and the one discovered by Finckler and Prior. These under the microscope look so much alike that one would not dare to differentiate between them, but their growth in gelatine tubes is so characteristic that one can easily tell the difference. The cholera bacillus grows upon the plate in a peculiar granular colony that does not make the gelatine fluid so quickly as the bacillus of Finckler or Deneke. Finckler makes the gelatine in tubes, fluid from above downward, first where the needle is run into it and again at the top. Those found in old cheese make it fluid in the same way, but less quickly, while the cholera bacilli grow in a characteristic funnel-shaped form. Then of the staphyolococcei, there is the styphylococcus pyogenes aureus, the staphyolococcus pyogenes citrius and the staphylococcus pyogenes albus; these are called white, orange and lemon color by their characteristic growth on culture media; *in time* one will be white, one orange and one lemon color; but in the first week one could not tell the difference. It is quite as important to know how to cultivate bacteria and detect the difference in growth as to know how to differentiate bacteria with the microscope by their form and their reaction to the analine dyes.

Dr. Lester Curtis read a paper on

### CALCAREOUS CONCRETIONS IN THE EXTERNAL EAR.

The following case, occurring in an old patient of mine, has seemed to me to be unusual. I had treated the patient for several years for slight ailments, chief of which was an occasional malarial attack that gave me some trouble on account of her inability to take quinine.

In August, 1886, she called my attention to a painful swelling in the external meatus of the right ear. I considered it to be a case of ordinary superficial inflammation of the meatus, and directed instillation of a solution of morphine. The pain at once ceased, and in a day or two the swelling subsided, and I supposed that the trouble had resolved, as has usually occurred in my experience after such treatment. But a little swelling and tenderness remained. In about two weeks, without further symptoms than a trifling uneasiness, there was a discharge of a drop of purulent fluid, which I am sorry that I had no opportunity of examining. I paid no more attention to the case until about a month after this, when she called at my office and told me that the same morning the ear had felt a little uncomfortable, and on picking it a calcareous mass about as large as a millet seed had come away. A day or two before she had removed a similar mass. These masses, she said, resembled lumps of old lime as nearly as anything she could think of. On examining the ear I saw a red and indurated place about half way down the meatus in the upper portion of the posterior wall. In the middle of this spot was a pit about as large as the mass removed, and surrounding the pit a greater or less amount of a white granular substance, which grated against the probe and was of stony hardness. I attempted to detach some of it with the sharpened end of the probe, but it was firmly adherent, and appeared to be infiltrated throughout the tissue, and I succeeded in getting away only a few small flakes. Besides this nothing unusual was detected in the ear with the exception of a slight redness of the posterior rim of the drum-head. The hearing was perfect. A slight feeling of irritation was noticed for some weeks, but gradually passed away. At present, so far as I can discover, the ear is quite normal.

I do not think that there is any possibilty of the concretion being a foreign body; nothing, with the exception of the morphine, was introduced into the ear, and that could hardly have produced such an effect; besides, part at least of the substance was infiltrated among the tissues of the meatus. Superficial abscesses, in which there is a fre e discharge of pus, do not undergo calcareous degeneration. The appearance was that of a gouty concretion, and there is a trace of gout in the family. Her father, though an active and abstemious man, having had slight attacks of the affection, but she has never had any gouty symptom that I have been able to discover, and gout does not suppurate. I have never heard of a case like this, and the somewhat limited amount of literature on diseases of the ear, accessible to me, makes no mention of anything of the kind.

Dr. R. Tilley thought the author should have examined the concretion microscopically and determine its nature. For himself he was of the opinion it was probably composed of dried epithelia resulting from a partial absorbed furuncle.

Dr. Lester Curtis: I have no special reply to make to the conjectures. I never examined the ear of the patient before this thing occurred, but I have examined both ears since that time, and so far as I could see there was nothing in the shape of a sebaceous cyst in either ear. One would naturally suppose that if such things occurred there would be more than one of them. I would imagine if one should occur in such a locality there would be likely to be others. One word in regard to treatment I used, which I have recommended in a considerable number of cases and always with benefit. While I have never used the rubber tube, I have seen a good many cases where I would not venture to insert it. The solution of morphine will quiet pain in every case; and in every case I have happened to meet, it has stopped the inflammation and the pain has not recurred.

Dr. W. L. Axford: I would suggest that the case was one originally of a subaceous cyst, that the contents of the cyst had undergone calcareous changes and afterwards inflamed. I have seen that happen in the head, but never in the ear.

Dr. W. L. Axford read a paper for Dr. O. G. Miller on

A GUIDE FOR THE OTIS URETHROTOME, in which the mechanism and workings of the guide were fully described.

Dr. R. Tilley reported a case of

ATHEROMA OF THE LEFT CORONARY ARTERY, RESULTING IN ANEURISM OF THE LEFT VENTRICLE.

Dr. Robert H. Babcock: It was my good fortune to see the patient to whom Dr. Tilley has referred this evening, and as it was a case of remarkable interest, and there are several points illustrative of the peculiar symptoms due to atheroma of the coronary arteries, and also illustrative of the difficulty, many times, of diagnosticating the condition I would like to refer to them. The first symptom mentioned by Dr. Tilley as important was that of angina pectoris, and herein this case was peculiar; the pains were flitting and were relieved by gentle exercise. It was wholly characteristic, however, in that the pain extended to the left shoulder and down the left arm. That is the characteristic pain of angina pectoris, although there are so many deviations from it that angina pectoris frequently passes unrecognized by the general practitioner. There is a liability oftentimes to mistake angina pectoris for neuralgia of the stomach. I have under observation a case where the pain has been considered for years gastralgia, but it is without doubt angina pectoris; the arteries are atheromatous and there are characteristic cardiac murmurs which are due to the atherosed condition of the valves. The pain of angina may sometimes be localized in the wrists. Dr. Sawyer once mentioned to me a case in which the angina pectoris always manifested itself by a feeling of cord-like constriction about each wrist. There was, however, with this an attendant paleness of the countenance and anxiety, and the patient was obliged to stop in his tracks until the pain had passed. The pain in angina pectoris may extend through to the back, or up into the occiput, at other times into the right shoulder and down the right arm. It may also radiate into the abdomen and down the inside of the thighs. It is usually so severe that the patient has to stand still and wait until the pain passes by. In the case mentioned the pain was relieved by gentle exercise, and that possibly may throw some light upon the question as to whether angina pectoris is always due to arterial spasm or not. If due to arterial spasm in all cases, it would seem as if any effort of the muscular system would increase the arterial resistance through contraction of the muscles, and thus make a greater demand upon the heart; in which case exercise ought to augment rather than relieve angina pectoris—particularly if, as Leyden is inclined to think, angina pectoris is really due to spasm of the coronary arteries as well as general arterial spasm, resulting in temporary anæmia of the heart muscle. In cases where the paleness of countenance would seem to confirm this theory, we find that remedies which produce arterial dilatation, such as the nitrate compounds, always give relief. In this case there is an apparent contradiction; not only did exercise afford relief which would seem to indicate that contraction of the peripheral vessels had nothing to do with the production of the angina, but, on the other hand, since nitro-glycerine gave relief, there must have been arterial spasm somewhere.

The diagnosis of this case was not as difficult as is often the case, owing to the peculiarity of the cardiac sounds, although none of us, I think, really suspected the condition which we found of the coagulum in the left ventricle filling up the lower third of the cavity. I gave my diagnosis of degeneration of the left ventricle, with atheroma of coronary arteries, because of the very peculiar character of the sound over the left ventricle which Dr. Tilley has very aptly described. It was a toneless, muffled sound as if the heart struck the chest wall through an intervening layer of cotton, while on passing the stethoscope from the left ventricle toward the right ventricle the first sound came out with its usual clearness and more or less of its booming quality, and it was on that that I based my diagnosis. At the post mortem examination we found degeneration of both ventricles, more marked in the left than in the right. This case would probably be classified by Leyden as subacute. Leyden has treated this subject in the most scientific manner of any author, since generally the subject is treated in a desultory way under the various heads of chronic myocarditis, fatty degeneration, aneurism, rupture of the heart, etc.

Leyden would group together all the different expressions of the same pathological condition, that is sclerosis of coronary arteries and all conditions dependent upon that, and he considers it under one subject of the changes of the heart resulting from the sclerosis of the coronary arteries. He recognizes as acute cases those in which the patient is ill perhaps for a few moments or hours or days. The first symptom is often a sharp attack of angina pectoris, or an attack of great cardiac distress and oppression without positive pain, which Gairdner speaks of as *angina sine dolore.* In these cases the patient frequently first discloses that he has heart disease by falling dead in his tracks. In cases of atheroma of a small branch of the left coronary artery, usually the anterior descending branch, frequently we find a condition closely resembling the hemorrhagic infarctus and the heart ruptures at that point; in acute cases death may be due to the rupture, although more often to failure of the heart. Subacute cases, of which this seems to be an example, run their course in a few weeks or months, and we find a combination of changes from acute fatty softening to chronic fibroid change, and these patients generally suffer a good deal with angina pectoris. The chronic cases are those that last for years, sometimes fifteen or twenty, and the symptoms are those of cardiac weakness or progressive cardiac failure, and these patients may die suddenly or gradually. In these cases we generally find extensive myocarditic changes with great thinning of the heart walls.

The treatment of these cases is of course a thing which interests us. It is very unsatisfactory; is merely palliative, and consists chiefly, in the severe cases, of rest, with cardiac tonics and stimulants. Of especial service in angina pectoris are the nitrite compounds, although Leyden rather protests against the use of nitrite of amyl and nitro-glycerine. He recommends the hypodermic injection of morphine, stimulants, such as ammonia and camphor, hot alcoholic drinks, and heat to extremities. The diagnosis has to be based, in most of these cases, upon the history and symptoms of progressive or sudden cardiac failure, and if we can find evidence of atheromatous change in the peripheral vessels the diagnosis is much easier. In auscultating these cases I have frequently found what seemed to me to be a peculiarity of the first sound. The sounds of the heart are nearly always described as free from murmurs, but since the booming quality of the first sound is undoubtedly due to the muscular element, and since the muscle of the heart is degenerated, the first sound takes on rather a short and valvular quality resembling very closely a second sound, so that frequently in these cases the two sounds appear to be two valvular sounds. Although I am not yet prepared to say that one can base a diagnosis upon such a condition as that, yet I am satisfied that in cases where there has been marked atheroma of the radial arteries, with symptoms, of heart failure, I have heard just this condition of shortness and valvular quality of the first sound over the left ventricle, particularly when over the right ventricle the sound is more prolonged.

Dr. Tilley said: I have nothing more to add, except that I have known of two or three cases in which, after a hypodermic injection of morphine, in a condition of atheromatous coronary arteries, the patient has passed off very rapidly, and I would be chary of using it, especially if I could obtain a satisfactory result from nitro-glycerine. The form of nitro-glycerine that I used were the elegant tablets of Fraser, so much more convenient than the alcoholic solution.

Dr. W. L. Axford: I have here the

SAC OF AN ANEURISM OF THE LEG, not very large or elaborate, but interesting because of its locality and the history of the case. Sometime last fall, along in November, a Bohemian came to the South Side Dispensary, having a little lump, about as large as a walnut, just above the internal malleolus. I could not get his history, as he could not talk English; all that I could make out was that it had been growing there about a year, and pained him some. The skin was natural over the growth, which felt soft and fluctuated. I made up my mind that it was some kind of a cyst, and advised him to have it excised. On cutting down upon the growth I found at once that I had made a mistake, from the color of the sac, which was blue. One of my assistants said he saw a little artery spurt. I stiched up the wound and found I had excised the sac of an aneurism. It was about the size of a walnut when fresh. This case was peculiar, as there were none of the usual symptoms of aneurism; it was also peculiar in the large size of the sac compared with the size of the artery. The patient made an uninterrupted recovery.

Dr. Jones, of Pittsburg, Pa.: I heard only a portion of the discussion on the subject of angina pectoris. One symptom I will mention as having occurred in my own experience that I believe is not mentioned in any of the literature that I am familiar with. In acute attacks of angina there is a feeling of constriction that may be relieved by firm pressure against a sharp body, such as the back of a chair or the edge of a table; that feeling of a patient wanting to press against some sharp substance, the back of a chair or the edge of a table, has been invariably present in all of the cases that I have seen, and I do not know that it is mentioned anywhere. The use of nitro-glycerine and amyl nitrite I would suppose would hardly be proper in chronic cases, on account of the danger spoken of, the danger of sudden cessation of the heart's action from paralysis or failure.

Dr. Lagree, of Charleston, S. C., mentioned some

### PECULIAR SYMPTOMS CAUSED BY EARTHQUAKES.

The earthquake in Charleston, with us, is a thing of the past. There were some peculiar features produced by the earthquake from a medical point of view. One of the most common conditions was an obstinate nausea, and in treating these cases we found the only remedy that would give any satisfactory result was muriate of cocaine. Another condition we found was falling off of the hair in patches, especially among the ladies. I have had three cases in which the hair gradually fell out in patches, which I attributed to nervous shock. Fortunately the hair was generally saved. Those were the most prominent symptoms produced by the earthquake.

